# Effect of a Warm-Up Protocol with and without Facemask-Use against COVID-19 on Cognitive Function: A Pilot, Randomized Counterbalanced, Cross-Sectional Study

**DOI:** 10.3390/ijerph18115885

**Published:** 2021-05-30

**Authors:** Maamer Slimani, Bianca Miarka, Hela Znazen, Wassim Moalla, Amri Hammami, Armin Paravlic, Nicola Luigi Bragazzi

**Affiliations:** 1 Department of Health Sciences (DISSAL), Postgraduate School of Public Health, Genoa University, 16132 Genoa, Italy; maamer2011@hotmail.fr; 2 Higher Institute of Sport and Physical Education of Ksar Said, University of Manouba, Manouba 2037, Tunisia; hammamiamri@hotmail.com; 3 Postgraduate Program in Physical Education, School of Physical Education and Sports, Federal University of Rio de Janeiro, Rio de Janeiro 21941-599, Brazil; miarkasport@hotmail.com; 4 Department of Physical Education and Sport, College of Education, Taif University, P. O. Box 11099, Taif 21944, Saudi Arabia; znazen.hela@laposte.net; 5 Higher Institute of Sport and Physical Education of Sfax, LR19JS01 EM2S: Éducation, Motricité, Sport et Santé, University of Sfax, Sfax 3000, Tunisia; wassim.moalla@gmail.com; 6 Science and Research Centre Koper, Institute for Kinesiology, Faculty of Sport, University of Ljubljana, 1000 Ljubljana, Slovenia; Armin.Paravlic@zrs-kp.si; 7 Laboratory for Industrial and Applied Mathematics (LIAM), Department of Mathematics and Statistics, York University, Toronto, ON M3J 1P3, Canada

**Keywords:** COVID-19, pandemic, coronavirus, facemask, exercise, neuropsychological tests

## Abstract

The present study aimed to verify the effect of a warm-up protocol with and without facemask-use on cognitive function. The sample was composed of 17 healthy, non-smoking physical education students (age = 17.6 years, height = 1.71 m, and body mass = 69.7 kg). They were randomized to perform 15 min of warm-up exercises, while wearing a cloth facemask (EXP) or no mask (CON) on two separate occasions, with at least 48-h separating conditions. Rate of perceived exertion (RPE) and d2 Attention assessment were used to verify cognitive function, using a repeated measures general linear model. The warm-up improved cognitive abilities and the results demonstrated significant differences between the EXP vs. CON groups in post-concentration performance (186.06 ± 15.47 EXP-score vs. 178.12 ± 13.66 CON-score), post the total number of errors (23.47 ± 14.50 EXP-frequency < 29.06 ± 13.74 CON-frequency), and in the post RPE (6.0 ± 1.37 EXP-index > 4.7 ± 0.85 CON-index). Wearing a cloth facemask caused positive effects on cognitive function. This data suggests that wearing a cloth facemask during warm-up may stimulate/improve the cognitive function.

## 1. Introduction

In late December 2019, a virus belonging to the *Coronaviridae* family, called the “Severe Acute Respiratory Syndrome-related Coronavirus type 2” (SARS-CoV-2) has emerged. A pneumonia outbreak was initially reported in the metropolitan city of Wuhan, in Hubei, mainland China, subsequently spreading to neighboring countries, and has gradually become a global pandemic. SARS-CoV-2 causes a generally asymptomatic or mild, but sometimes severe and even life-threatening respiratory disease, termed as “Coronavirus Disease 2019” (COVID-19) [1].

The still ongoing pandemic represents a major public health challenge, due to the highly contagious, quickly spreading nature of the virus, which has been overwhelming for healthcare settings and facilities, globally. These are facing shortage of personnel and medical equipment, which has further increased their strain. Vaccines have been licensed and approved only recently, which has resulted in the implementation of non-pharmaceutical interventions (NPIs), including enhanced hygiene practices, wearing of masks, practicing of social distancing, self-isolation, quarantine, and partial/total lockdowns [2].

Some groups are particularly vulnerable and prone to SARS-CoV-2, including the frail elderly, who are at a higher risk for contracting the virus and developing complications. Physical activity and exercise have been proposed to counteract, or at least, mitigate the burden imposed by the virus, as well as the detrimental effects of NPIs on mental and physical health and general well-being [3,4,5].

However, maintaining adequate physical activities levels during the COVID-19 pandemic is challenging. Even though the use of a protective facemask is compulsory in shared and confined environments for individuals aged 10 years and older, the World Health Organization (WHO) recommends against wearing a mask while exercising [6] due to the fact that the mask could impact breathing capacity. Exercise and physical activities are recommended outdoors, whereas if practiced indoors, social distancing should be respected, while ensuring adequate ventilation. If it is unfeasible to observe these protocols, physical activities should be temporarily cancelled/postponed or practiced while wearing a protective facemask with an appropriate intensity [7]. 

Few research has been carried out on the benefits and the disadvantages of wearing medical, non-medical, and other protective facemasks during the practice of a physical activity. Some studies have shown statistically significant impaired physiological cardio-pulmonary parameters in healthy subjects, and individuals suffering from pre-existent moderate-to-severe respiratory afflictions (including asthma or chronic obstructive broncho-pneumopathy, COPD), during a light-to-moderate physical activity [8,9,10,11,12,13,14]. Changes of face temperature, moisture, and micro-climate, together with perceived dyspnea have been documented in some studies [15]. However, the existing scholarly literature reports contrasting findings, with a recently published review showing no evidence of detrimental effects in healthy subjects exercising while wearing masks, whereas some concerns were raised among patients with severe cardio-pulmonary conditions [7].

To the best of our knowledge, there is a dearth of data concerning the impact of practicing physical activity, while wearing a protective facemask, on cognitive function. Therefore, the present study was conducted to fill in this gap in knowledge. The aim of the present study was to verify the effect of a warm-up protocol, with and without facemask use, on cognitive function.

## 2. Materials and Methods

### 2.1. Participants

Seventeen healthy (9 males and 8 females), non-smoking, physical education students (age = 17.6 years, height = 1.71 m, and body mass = 69.7 kg) volunteered to participate in this study after being informed of the nature and of the possible risks associated with the experiment. They were randomized to perform warm-up, while wearing the same cloth facemask (EXP) or no mask (CON) on two separate occasions, with at least 48-h separating the two conditions in order to allow the participants a full recovery [16]. All participants recovered for the same amount of time. Each session took place at approximately the same time of the day (± 1.5 h). We only included participants who (a) were active and healthy; (b) without co-morbidities like diabetes, hypertension, epilepsy, cardiac illness, asthma, and other respiratory illness; and (c) were inactive for 48 h before the physical activity session.

The cloth mask (half and quarter masks, Tunisia) was a 3-layer comfortable elastic and extra-soft ear-loop, so as to eliminate pressure to the ears, with layers composed of non-woven fabric, and was considered to be a facemask that is typically used by the general population [12]. All participants wore the same facemask brand/type.

This study was conducted in Tunisia and its findings were reported according to the “Consolidated Standards of Reporting Trials” (CONSORT) guidelines. 

### 2.2. Protocols

In this randomized counterbalanced, cross-sectional study, participants performed 15-min of warm-up exercises. After light tempo runs (4 min), the participants performed arm circles, jumping jacks, high knees jog, and back kicking (2.5 min). Then, this warm-up was followed by some stretching exercises (1.5 min). Participants continued the warm-up protocol by performing 2 sets of each exercise in static and dynamic manners—push-up, sit-up, and squat movements (30 s of work and 30 s of rest). 

#### 2.2.1. Attention Assessment

The d2 test was used to determine the level of concentrated visual attention of participants [17]. It consists of 14 rows with 47 characters per line. These characters are the letters d or p, with a total of one to four dashes above and below each letter. Participants were asked to scan each line and cross out only the characters containing the letter d with two dashes during 20 s.

After completion of the d2 test, two variables were calculated—concentration performance (CP) and total number of errors made by the participants (E). CP is calculated as the number of correctly marked d2-symbols minus the number of incorrectly marked symbols (symbols that are not d2-symbols). The total number of E was assessed as the number of errors made by participants by failing to correctly identify a d2-symbol plus the number of errors made by incorrectly marking symbols that were not d2- symbols. This tool was tested/validated in a previous publication [18].

#### 2.2.2. Rating of Perceived Exertion (RPE)

Before and after each session, the “Rating of Perceived Exertion” (RPE) scale was used to estimate the participants’ perceived effort. It ranged between 0—“no perceived effort” (i.e., rest) and 10—“maximal perceived effort” (i.e., the most stressful exercise ever performed) [19].

### 2.3. Statistical Analysis

Descriptive statistical analysis was carried out by computing the means and standard deviations for each of the variables under study. Normality of data distribution was verified by applying the Shapiro-Wilk test, which was preferred over other normality tests, given the sample size employed in the present investigation. Main effects were studied with a repeated measures General Linear Model (GLM), with condition (wearing mask or not) and time (PRE and POST) as within factors. The magnitude of difference was measured by using Cohen’s d and was interpreted as follows—trivial: 0.0–0.2; small: 0.2–0.5; moderate: 0.5–0.8; and large: ≥ 0.8. All statistical analyses were conducted utilizing the commercial software “Statistical Package for Social Sciences” (SPSS version 24.0, IBM, Armonk, NY, USA). Results with *p*-values less than or equal to 0.05 were considered to be statistically significant.

## 3. Results

Seventeen subjects conducted the assessment of cognitive function before and after the warm-up protocol. Regardless of the condition they were allocated to, significant alterations were observed for all measures of interest (all, *p* < 0.001), following the warm-up protocol.

### 3.1. Concentration Performance

Both EXP and CON groups had significantly higher CP following a warm-up protocol, as compared to PRE (F_1,32_ = 115.239, *p* < 0.001, η^2^ = 0.783) (Table 1). There was a significant time × group interaction effect (F_1,32_ = 5.671, *p* = 0.023, η^2^ = 0.151). Post-hoc analysis showed that the EXP group experienced a significantly higher increase in CP, as compared to the CON group (+8%, *p* = 0.023).

### 3.2. Total Number of Errors

Both EXP and CON groups had significantly lower E following a warm-up protocol, as compared to PRE (F_1,32_ = 78.924, *p* < 0.001, η^2^ = 0.712), however, without a significant time × group interaction effect (F_1,32_ = 3.029, *p* = 0.091, η^2^ = 0.086) (Table 1). Post-hoc analysis showed that the EXP group had less errors compared to the CON group, however, the observed difference was not significant (−15%, *p* = 0.091).

### 3.3. Rate of Perceived Exertion

Both EXP and CON groups had significantly higher RPE following a warm-up protocol, as compared to PRE (F_1,32_ = 647.063, *p* < 0.001, η^2^ = 0.953). There was a significant time × group interaction effect (F_1,32_ = 7.063, *p* = 0.012, η^2^ = 0.181) (Table 1). Post-hoc analysis showed that the EXP group experienced significantly higher increase in RPE compared to the CON group (+36%, *p* = 0.013).

## 4. Discussion

In the present work, we found statistically significant differences in terms of concentration performance and RPE, between the experimental and control groups, before and after a warm-up protocol. Differences in total number of E could be detected before and after, but failed to achieve the statistical significance threshold, when stratifying the analysis based on the allocated group.

In the current investigation, a well-developed warm-up protocol was designed and implemented. Warm-up precedes the practices of almost every athletic competition, in order to enhance the readiness for subsequent exercise and performance. Typically, stretching, technical practice, and activities of varying intensity are used as fundamental components of the warm-up protocol and are usually completed within 10–15 min. The warm-up aims to favor a smooth transition of the athlete from a state of rest to a state of exercise, while reducing residual fatigue. Therefore, it is not surprising that 79% of studies investigating the effects of warm-up practices on subsequent physical performance had observed improvements, in terms of performance outcomes [20]. Furthermore, with regards to the effect of warm-up on cognitive function, our study corroborates Elsworthy et al.’s study [21]. They found that concentration performance and reaction time were significantly improved (*p* < 0.001) following the physical warm-up protocol, by approximately 8%. On the other hand, attention and concentration did not differ according to the type of performed warm-up [22]. Due to the positive acute effects of exercise on attention and physical performance showed in the above-mentioned studies, we could suggest that a well-prepared warm-up is beneficial for preparing the athletes to the following activities, especially competition. However, to the best of the authors’ knowledge, the results published in the existing scholarly literature are generally contradictory [21,22,23,24,25]. For instance, Budde et al. [23] showed a significant improvement of visual selective attention, and the ability to concentrate, after a 10-min coordination exercise in adolescents. Additionally, a 20-min treadmill exercise at 60–70% HR_max_ resulted in an improvement of accuracy with no effects on reaction time [24]. However, the impact of exercise on cognition maybe more subtle and complex than just a linear association. In their recent review, Janssen et al. [25] concluded that an inverted “U” relationship can be found between exercise intensity and its acute effects on attention. When the intensity is too high or too low, the positive impact on attention is lost. Due to the lack of a sufficient number of studies and given the discrepancy of the results, it is very important to continue experimenting the effects of the warm-up on cognitive performance with different parameters, such as training modalities (volume, intensity, and frequency), teaching style, stimuli, etc. However, according to the review of Janssen et al. [25], we could assume that the positive effects we had noticed could depend on the length and intensity of the intervention.

Facemask-use confers protection against SARS-CoV-2, and, together with other implemented NPIs, it has contributed to curbing the burden imposed by the COVID-19 pandemic. However, exercising with protective facemasks may be accompanied by physiopathological side-effects that could significantly counteract the beneficial impact of the mask itself. Carbon dioxide exchange could indeed be impaired due to the potential decrease in available oxygen and the increase in air trapping. This would result in hypercapnic hypoxia, which in turn, may lead to an increased acidic environment, anaerobic metabolism, and cardiac and renal overload [26]. From a motor-cognitive perspective, this is expected to impact cognitive functions [27]. However, some empirical studies seem to disconfirm the hypothesis of the hypercapnic hypoxia. For example, in a sample of 50 adults (one-third of which has an underlying co-morbidity), no episodes of hypoxemia or hypercarbia occurred. No differences in CO_2_ or oxygen saturation (SpO_2_) could be detected between baseline values and those measured while exercising (walking briskly for ten minutes) when wearing a mask [28].

The findings of the present investigation, in corroborating the confutation of the hypercapnic hypoxia hypothesis, complement and add to the works of previous authors [15,29,30]. For instance, Morris et al. [29] studied the effects of prolonged facemask-use during 45 min of light exercise on motor-cognitive performances, in a sample of 8 participants. The authors showed that motor-cognitive performance did not differ between prolonged facemask-use and the control condition. Similar findings were reported by another study, which investigated the effect of surgical-mask-use on cognitive and psychophysiological response, in a sample of biomedical students, after a 150-min lesson [30]. The authors showed that surgical-mask-use had no impact on reaction time. While we found a positive impact of wearing facemasks on cognitive functions, the studies previously mentioned did not report any detrimental effects, but also could not detect benefits in terms of cognitive gains. These differences could be explained by the methodology adopted, such as the length of the intervention (15 versus 45/150 min) and the type/intensity of physical activities performed. Inversely, few studies found that wearing facemasks interfered with cognitive and executive tasks such as face recognition and matching [31].

Furthermore, we found that both groups (EXP and CON) showed different results for RPE, which was not in line with the scholarly literature. For example, Morris et al. [28] showed that physiological strain or thermal discomfort did not differ between prolonged facemask-use and the control condition. Only perceived dyspnea was found to significantly differ between the two conditions. Similarly, another study [30] showed that surgical mask had no impact on mental fatigue perception. On the contrary, Li et al. [15] recruited a sample of five healthy male and five healthy female participants, carrying out intermittent exercise on a treadmill. Participants wore facemasks and exercised in a controlled climate chamber (air temperature of 25 °C, relative humidity of 70%). Several types of protective facemasks were compared—namely, N95 (3M 8210) and surgical facemasks, either treated or not with nano-functional materials. The use of surgical masks correlated with lower mean heart rates, micro-climate, skin temperature, absolute humidity, breath resistance, overall discomfort, and fatigue. Subjects expressed their preference for nano-treated versus untreated surgical facemasks. On the other hand, in another setting, Garra et al. [32] conducted a non-randomized, hospital-based cohort study recruiting a sample of 144 healthcare personnel working at a single tertiary center. The authors evaluated whether wearing a medical mask or the combination of a medical mask and N95 could impact routine clinical activities. No differences could be found between the two groups, in terms of mental and physical fatigue, yawning, and headache development. 

Finally, our investigation should be considered to be a pilot study that explores the effects of wearing a facemask while exercising on cognition. Even though this study used randomized trials, the limited sample size is a limitation and warrants further studies. Future studies could aim to replicate our findings in a more statistically robust fashion, and investigate the impact of variables such as type of warm-up protocol, its length, and intensity, as well as type/brand of facemask employed. 

## 5. Conclusions

Our main results revealed the positive effect of the facemask use when practicing a warm-up on concentration performance. These data suggest that wearing a cloth facemask during warm-up may stimulate the cognitive function. More studies on masks’ material for sports practice are crucial for warm-up activities and specific sports practice. Future research should investigate the effect of acute exercise, as studies exploring different frequency and intensity on cognitive function are highly needed.

## Figures and Tables

**Table 1:** Comparison of cognitive function assessment between those who wore the facemask and those who did not, following a warm-up protocol.

Parameters		With Face Mask (EXP: N = 17)		Cohen’s D	Without Face Mask (CON: N = 17)		Cohen’s D	Main Effect	Interactions
		Mean	SD		Mean	SD		*p*-value [η^2^]	*p* value [η^2^]
Concentration performance	PRE	155.12	23.240		158.41	19.701			
	POST	186.06	15.470	1.3	178.12	13.665	1.0	**<0.001 [0.783]**	**0.023 [0.151]**
Total number of errors	PRE	54.35	26.498		49.82	20.415			
	POST	23.47	14.496	−1.2	29.06	13.736	−1.0	**<0.001 [0.712]**	0.091 [0.086]
Rate of perceived exertion	PRE	1.65	0.931		1.18	0.883			
	POST	6.00	1.369	4.7	4.71	0.849	4.0	**<0.001 [0.953]**	**0.012 [0.181]**

η^2^—partial eta-squared; bold values—statistical significance.

## Data Availability

All data generated are available within the present manuscript.
